# IL-17 Affects the Progression, Metastasis, and Recurrence of Laryngeal Cancer *via* the Inhibition of Apoptosis through Activation of the PI3K/AKT/FAS/FASL Pathways

**DOI:** 10.1155/2020/2953191

**Published:** 2020-12-18

**Authors:** Yang Song, Ming Yang, Hongjian Zhang, Yan Sun, Ye Tao, Huihui Li, Jing Zhang, Yuncheng Li, Jianming Yang

**Affiliations:** ^1^Department of Otorhinolaryngology, The Second Hospital of Anhui Medical University, Hefei, Anhui 230601, China; ^2^Department of Otorhinolaryngology and Head and Neck Surgery, Yuhuangding Hospital of Qingdao University, Yantai, Shandong 264000, China; ^3^Department of Otolaryngology-Head and Neck Surgery, Beijing Tongren Hospital, Capital Medical University, Key Laboratory of Otolaryngology Head and Neck Surgery, Beijing 100730, China; ^4^Physical Examination Center, Union Hospital, Tongji Medical College, Huazhong University of Science and Technology, Wuhan, Hubei 430022, China; ^5^Department of Otorhinolaryngology, Union Hospital, Tongji Medical College, Huazhong University of Science and Technology, Wuhan, Hubei 430022, China

## Abstract

**Background:**

Cytokines play important roles in the development and prognosis of laryngeal cancer (LC). Interleukin-17 (IL-17) from a distinct subset of CD4+ T cells may significantly induce cancer-elicited inflammation to prevent tumor immune surveillance.

**Methods:**

The expression levels of IL-17 were examined among 60 patients with LC. Immunofluorescence colocalization experiments were performed to verify the localization of IL-17 and FAS/FASL in Hep-2 and Tu212 cells. The role of IL-17 was determined using *siRNA* techniques in the LC cell line.

**Results:**

In the LC patients, cytokines were dysregulated in LC tissues compared with normal tissues. It was found that IL-17 was overexpressed in a cohort of 60 LC tumors paired with nontumor tissues. Moreover, high IL-17 expression was significantly associated with the advanced T category, the late clinical stage, differentiation, lymph node metastasis, and recurrence. In addition, the time course expression of FAS and FASL was observed after stimulation and treatment with the IL-17 stimulator. Finally, *in vitro* experiments demonstrated that IL-17 functioned as an oncogene by inhibiting the apoptosis of LC cells *via* the PI3K/AKT/FAS/FASL pathways.

**Conclusions:**

In summary, these findings demonstrated for the first time the role of IL-17 as a tumor promoter and a prometastatic factor in LC and indicated that IL-17 may have an oncogenic role and serve as a potential prognostic biomarker and therapeutic target in LC.

## 1. Background

The development of laryngeal carcinoma (LC) has increased recently and is the second most common malignant tumor of the head and neck. It is the sixth most common tumor worldwide, with a five-year survival rate of approximately 50% [1]. Despite advances in treatment including surgery, radiotherapy, and chemotherapy or their combination, adjuvant treatment with chemotherapy and molecular targeting is emerging as a more effective therapeutic option for advanced LC [2, 3].

IL-17 (IL-17A) is one of the members of the IL-17 family, and the IL-17 family includes IL-17A, IL-17B, IL-17C, IL-17D, IL-17E (IL-25), and IL-17F. The IL-17F has the highest degree of homology to IL-17A in the family. T helper cell 17-secreting cells (Th17) are the primary source of IL-17, while other cell types also develop this cytokine, such as group 3 innate lymphoid cells (ILC3), *δγ*T cells, and natural killer (NK) cells [4–6]. Some evidence has suggested that IL-17 is a key proinflammatory cytokine for the induction of cytokine and chemokine secretions by other cell types, among which mesenchymal cells and myeloid cells can recruit monocytes and neutrophils for inflammation [7, 8]. In addition, much evidence has demonstrated that IL-17 markedly causes tumor growth and angiogenesis, indicating IL-17 plays a role in tumor promotion [9]. IL-17 has been shown to activate the Src/PI3K/AKT/nuclear factor-*κ*B (NF-*κ*B), MAPK, Stat3, and Cox-2 pathways, which play significant roles in tumorigenesis, angiogenesis, and metastasis [9, 10]. Studies have also shown that IL-17 was found to be overexpressed in some human tumors, such as cervical cancer, breast cancer, gastric cancer, colorectal cancer, and other cancers [11–17].

FAS, a type I membrane protein, transmits a suicide signal to the cell, binds to its ligand (FALL) or anti-FAS antibodies, and leads to caspase-8-dependent cell death [18, 19]. The PI3K/AKT pathway plays an important role in the regulation of cellular processes, which control cell size/growth, proliferation, survival, glucose metabolism, genome stability, and neovascularization. Some studies have demonstrated that Forkhead box O3 (also known as FOXO3) inhibited the FALL gene promoter [20, 21]. Furthermore, AKT-mediated phosphorylation of FOXO3 more likely favors cellular survival *via* enhancing the retention of FOXO3 in the cytoplasm [22, 23].

Given the roles of IL-17 in transducing multiple signals in cells, IL-17 also activates Src to promote cancer development [6]. For example, the PI3K/AKT signaling pathway is one of the major Src-activating pathways and may block the FAS-associated death domain protein to inhibit cellular apoptosis [24, 25]. Thus, it is likely that IL-17 exerts its function through the FAS and FASL signaling pathways [26, 27]. To date, little is known about the roles of IL-17 in LC, and the regulation of IL-17 in LC has not been fully investigated. This study is aimed at characterizing its function in LC both *in vitro* and *in vivo*.

## 2. Methods

### 2.1. Study Patients

A total of 60 patients (ages 22 to 88 years old, 35 female cases, and 25 male cases), who were pathologically diagnosed with laryngeal carcinoma from January 2014 to December 2017 from the Second Hospital of Anhui Medical University, were included in this study. The patients' tumor tissues were collected from several groups with different degrees of differentiation. All of the patients had received surgical treatment without radiotherapy, chemotherapy, or molecular targets prior to their operation and conventional imaging examinations (CT, MRI). Laryngeal tumor tissues and matched adjacent tissues were surgically removed and immediately collected by research assistants. These specimen samples were obtained from at least 5 mm from the tumor margin and were stored at −80°C until use. Thus, all of these patients received surgery only and were confirmed with LC using pathological examination at the Second Hospital of Anhui Medical University. All of the patients in this study met the criteria of the World Health Organization (WHO) Histological Classification of Tumors of the Gallbladder (2003). Tumor staging was assessed according to the TNM staging guidelines by the International Union Against Cancer (UICC). Signed informed consent forms were obtained from each patient, and the study was approved by the institutional research board of the ethical committee at the Second Hospital of Anhui Medical University.

### 2.2. Quantitative Real-Time PCR

The total RNA from LC tissues and LC cells were isolated using the TRIzol reagent. The total RNA was extracted using the TRIzol reagent (Invitrogen, Carlsbad, CA). PrimeScript™ RT Reagent Kit with gDNA Eraser (Takara, Kusatsu, Japan) and Mir-X™ miRNA First-Strand Synthesis Kit (Takara) were used to synthesize the cDNA from the mRNA and miR, respectively. The qRT-PCR was performed using the iQ5 Real-Time PCR System (Bio-Rad, Hercules, CA, USA) with the SYBR Premix Ex Taq™ (Takara, Japan). The cycle threshold value was defined as the PCR cycle number at which the reporter fluorescence crossed the threshold. The cycle threshold value of each product was determined and normalized against that of the internal control. All measurements were performed in triplicate. Data were analyzed using the 2-*ΔΔ*CT method (Table 1).

### 2.3. Cell Culture

*In vitro* studies were performed on immortalized human laryngeal epidermoid carcinoma (Hep-2) cells and human laryngeal carcinoma (Tu212) cells obtained from the American Type Culture Collection. Recombinant Human IL-17A (200-17) was the product of the PeproTech Company (PeproTech, Hamburg, Germany). Z-IETD-FMK was obtained from Abcam (Cambridge, MA, USA). The PI3K inhibitor, wortmannin, was purchased from the Beyotime Institute of Biotechnology (Shanghai, China). All cells were incubated at 37°C with 5% CO_2_ in Dulbecco's Modified Eagle's Medium (DMEM) containing 10% fetal calf serum (FCS), 2 mmol/L L-glutamine, and 5000 IU/mL penicillin/5000 g/mL streptomycin for 1 to 2 days before beginning the experiments.

### 2.4. Cell Transfection

Hep-2 cells and Tu212 cells were cultured in 60 mm plates or 100 mm plates (for Western blotting analysis) without antibiotics overnight. When the cells reached 70–80% confluency, they were transiently transfected with IL-17 siRNA and scramble siRNA (GenePharma, Shanghai, China) using Lipofectamine RNAiMAX (add the source) according to the manufacturer's recommendations.

### 2.5. Flow Cytometry

The cells at the logarithmic phase were inoculated into a fresh culture medium and cultured in a 37°C and 5% CO_2_ incubator for 48 hours. The cells were divided into two groups: a blank control group with normal cells without treatment and an IL-17 siRNA group with transfection of IL-17 siRNA. Then, the cells were digested with trypsin to obtain a cell suspension, which was centrifuged at 1000 rpm for 5 min to allow for the collection of the cells. After the cells were washed with precooled phosphate-buffered solution (PBS) twice, the cells were resuspended in 500 *μ*L of the binding buffer at a concentration of 10^6^ cells/mL and then mixed with 10 *μ*L of Annexin V-FITC (Beyotime Institute of Biotechnology, Shanghai, China) for 15 min in the darkroom at room temperature. Then, 5 *μ*L of propidium iodide (Beyotime Institute of Biotechnology, Shanghai, China) was added to the cells. After incubation for 5 min in the darkroom at room temperature, the samples were analyzed using a FACSAria flow cytometer according to the manufacturer's instructions (BD Biosciences, San Diego, CA, USA).

### 2.6. Immunofluorescence Colocalization Assay

For the fluorescence staining, the cells were fixed with 40 g/L formaldehyde, permeabilized with 0.1% Triton X-100 in PBS, and blocked with 1% bovine serum albumin (BSA) in PBS for 30 min. This was followed by incubation overnight at 4°C with both anti-flag and anti-RhoC antibodies. The cells were washed three times with PBS for 5 min, incubated with DyLight™ 488 conjugated Goat anti-Mouse IgG along with DyLight™ 549 conjugated Goat anti-Rabbit IgG for 30 min, and then nuclear stained using 1 mg/L 4′,6-diamidino-2-phenylindole (DAPI, Roche, Germany). The fluorescence images were acquired with an Olympus FV1000 confocal microscope (Olympus, Japan) using a 100x oil immersion objective.

### 2.7. TUNEL

The cells were cultured on coverslips in six-well plates. The Hep-2 cells and Tu212 cells were plated in an 18 mm covered glass with McCoy's 5A at ∼70% confluence and incubated for 24 h at 37°C. The cells were divided into three groups. The blank control group had normal cells without treatment; the scramble group was transfected with scramble siRNA; and the IL-17 siRNA group was transfected with IL-17 siRNA. The cells were then treated with Rh2 for 48 h. After that, the cells were fixed in ice-cold 2% paraformaldehyde (PFA), washed with PBS, and stained with the TdT-mediated dUTP Nick-End Labeling (TUNEL) Kit. The TUNEL was subsequently performed using the *In Situ* Cell Death Detection Kit (Roche, Penzberg, Germany) following the manufacturer's instructions. The nucleus stained by DAPI was blue under ultraviolet excitation, and the positive apoptotic nucleus was stained in green. The percentage of the number of TUNEL positive cells were then analyzed on a flow cytometer (BD FACSAria) with the ImageJ software according to the manufacturer's instructions (BD Biosciences, San Diego, CA, USA).

### 2.8. Western Blotting

Laryngeal carcinoma tissue and paracancerous tissue samples were excised during operation and flash-frozen at −80°C until use. In order to prepare lysates, frozen laryngeal carcinoma tissues were minced with eye scissors on ice. Then, they were homogenized in a lysis buffer (1% sodium deoxycholate, 1% sodium dodecyl sulfate (SDS), 1% Triton X-100, 1% NP-40, pH 7.5, 5 mmol/L EDTA, 50 mmol/L Tris, 1 *μ*g/mL leupeptin, 10 *μ*g/mL aprotinin, and 1 mmol/L PMSF) and centrifuged at 12,000 rpm at 4°C for 20 min to collect the supernatant. Cell cultures for immunoblotting were lysed with a sodium lauryl sulfate loading buffer and stored at −80°C until use. Protein concentration was then quantified by a BCA assay using the Pierce™ BCA Protein Assay Kit (Thermo Fisher Scientific Inc.) according to the manufacturer's instructions. The collected supernatant was subjected to SDS-polyacrylamide gel electrophoresis (SDS-PAGE) after determining the protein concentration using the Bradford assay (Bio-Rad). After the separated proteins were transferred to polyvinylidene difluoride filter (PVDF) membranes, the membranes were blocked with 1% (*w*/*v*) bovine serum albumin (BSA) for 2 h and incubated with primary antibodies against IL-17 (anti-rabbit, 1 : 1000; Abcam), IL-17R (anti-mouse, 1 : 1000; Sigma), FAS (anti-rabbit, 1 : 1000; Cell Signaling), FASL (anti-rabbit, 1 : 1000; Cell Signaling), and *β*-actin (anti-rabbit, 1 : 1000; Abgent) at 4°C overnight. Finally, the membranes were incubated using the second antibody at 37°C for 2 h. Bound proteins were scanned using ChemiDoc XRS (Hercules, CA).

### 2.9. Statistical Analysis

The data were expressed as the means ± standard deviations. The values in the tables and figures are given as means and standard deviations of the means if not otherwise indicated. The analysis of variance (ANOVA) and Student's *t*-test were used in the SPSS software to determine the significant differences between groups. A *χ*^2^ test was used to evaluate the associations between clinicopathologic variables and IL-17 protein expression. The correlation between the mRNA levels of IL-17 and FAS and FASL in the LC tissue was analyzed using Pearson's correlation analysis.

All of the patients signed informed consent forms upon admission to the hospital. The end outcome of the study was set as disease recurrence. For the follow-up, the LC patients were either typically followed and monitored through their treatment and posttreatment courses with regularly scheduled clinical and radiographic examinations or retrospectively obtained from the patients' relatives by either phone or mail during the follow-up. Time to recurrence was computed from the date of end of treatment to the date of last follow-up or the date of clinically detectable recurrent cancer (local, regional, or distant). Participants who were recurrence-free or lost to follow-up were considered censored.

The Kaplan-Meier survival analysis and log-rank tests were used to calculate the survival curves. The Cox proportional hazard regression model was used to estimate overall survival. The best cut-off value for IL-17 expression was determined based on the receiver operating characteristic (ROC) analysis. Values of *P* less than 0.01 were considered to be statistically significant. The data were analyzed using the SPSS 20.0 statistical software program (version 20.0; IBM Corporation, Armonk, NY, USA).

## 3. Results

### 3.1. Association of Increased IL-17 Expression with Tumor Progression in LC Patients

**Figure 1:**
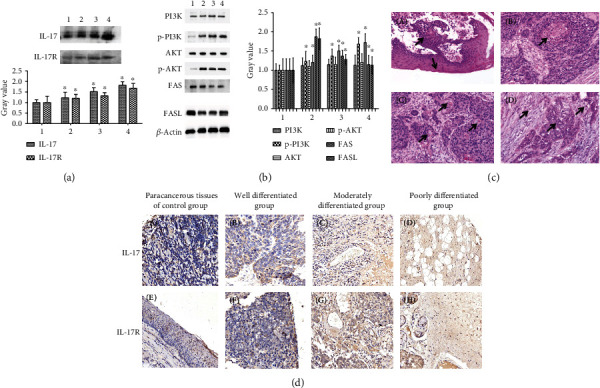
Western blotting analysis was used for time course analysis of IL-17, IL-17R, P-AKT, P-PI3K, FAS, and FASL protein expression in different pathological grades of LC tissues. (a) IL-17 and IL-17R protein expression in paracancerous tissues of the control group and different pathological grades of LC tissues. (b) P-AKT, P-PI3K, FAS, and FASL protein expression in paracancerous tissues of the normal control group and different pathological grades of LC tissues. The bar graph of protein quantification plotted from not <3 independent experiments. The band intensity of IL-17, IL-17R, P-AKT, P-PI3K, FAS, and FASL was quantified by densitometry and normalized to *β*-actin. Densitometry values in the histograms were expressed as fold change relative to the normal control, which was assigned a value of 1. ^∗^*P* < 0.01 compared with the control group. 1 = paracancerous tissues of the control group, 2 = well-differentiated group, 3 = moderately differentiated group, and 4 = poorly differentiated group. LC: laryngeal cancer. (c) LC, lymphocytic host response, and tumor infiltrative lymphocytes. (A) Arrows show paracancerous tissues of the control group with pushing invasive tumor front and band-like dense inflammatory infiltrates. H&E,400x. (B) Arrows show the well-differentiated group demonstrating minimal lymphocytic host response. H&E, 400x. (C) Arrows show the moderately differentiated group with a more intense lymphocytic host response. H&E, 400x. (D) Arrows show tumor-infiltrating lymphocytes in the poorly differentiated group. H&E, 400x. H&E: hematoxylin and eosin. (d) IHC analysis of IL-17 and IL-17R protein expression levels in LC tissues from patients at different pathologic grading. The (A–D) indicated IL-17 expression in LC tissues from patients with different pathologic grading. The (E–H) indicated IL-17R expression in LC tissues from patients with different pathologic grading. Scale bar = 50 *μ*m. ^∗^*P* ≤ 0.05.

**Figure 2:**
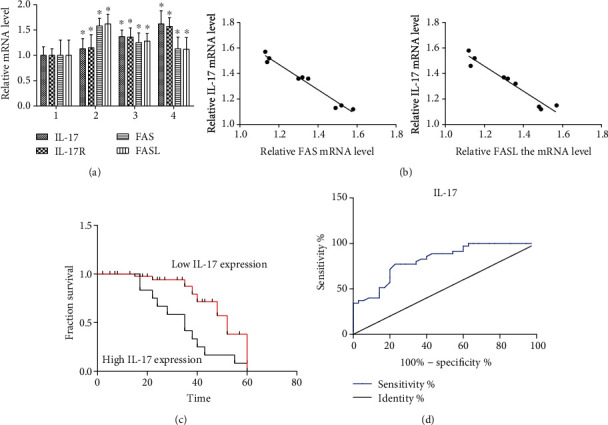
Relative expression of IL-17, IL-17R, FAS, and FASL at the mRNA level in paracancerous tissues of the control group and different pathological grades of LC tissues. One representative experiment of the three independent experiments is demonstrated. ^∗^*P* < 0.01 compared with the control group. 1 = paracancerous tissues of the control group, 2 = well-differentiated group, 3 = moderately differentiated group, and 4 = poorly differentiated group. (a) IL-17, IL-17R, FAS, and FASL expression was determined using the reverse transcription-quantitative polymerase chain reaction. (b) The scatter plot of IL-17, FAS, and FASL expression revealed a strong negative correlation, indicating that IL-17 expression was correlated with the apoptosis in LC cells (*r* = −0.82 for FAS and *r* = −0.847 for FASL, and all *P* < 0.01). (c) Disease-free survival by IL-17 expression in LC patients (*n* = 60). (d) The ROC curves and AUC values of IL-17 expression (AUC = 0.8184, 95% CI: 0.721-0.915, and *P* < 0.0001). Expression of the IL-17 and IL-17R proteins in LC tissues from patients at different pathologic grading.

As shown in Figure 1(a), the Western blotting in the different pathological grades of LC tissues showed that the expression levels of the IL-17 and IL-17R proteins increased significantly with increasing severity of LC. These findings were consistent with the P-AKT and P-PI3K levels of protein expression, while the expression of FAS and FASL in the LC tissues decreased significantly. The expression of PI3K and AKT had no significant expression changes compared with the paracancerous tissues of the normal control group (Figure 1(b)). In the hematoxylin and eosin assay (Figure 1(c)), paracancerous tissues demonstrated keratosis and superficially invasive LSCC with a pushing invasive tumor. Additionally, front and band-like dense inflammatory infiltrates (Figure 1(c), A), well-differentiated tissues that demonstrated tumor-infiltrating lymphocytes in nonkeratinizing LSCC (Figure 1(c), B), moderately differentiated tissues that showed keratinizing invasive LC with a more intense lymphocytic host response (Figure 1(c), C), and superficially invasive keratinizing LC of the poorly differentiated tissues demonstrated a minimal lymphocytic host response (Figure 1(c), D). To confirm whether IL-17 and IL-17R protein overexpression was correlated with LC pathologic grading, the IHC was performed on 60 LC tissue samples which included paracancerous tissues of the normal control group (10 cases), well-differentiated group (12 cases), moderately differentiated group (22 cases), and poorly differentiated group (16 cases). We found that 28 samples (46.7%) for IL-17 and 27 samples (45%) for IL-17R had high protein expression. These results were consistent with the results of Western blotting as shown in Figure 1(d). The hematoxylin and eosin assay demonstrated more lymphocytic infiltration with a decrease in the degree of differentiation in LC. Moreover, the mRNA expression levels of IL-17, IL-17R, FAS, and FASL were determined using qRT-PCR in different pathological grades of LC tissues in the well-differentiated, moderately differentiated, and poorly differentiated groups. The mRNA expressions of IL-17 and IL-17R were significantly increased in LC tissues, while FAS and FASL decreased markedly with the decreased degree of differentiation (Figure 2(a)).

### 3.2. Correlation Analysis of IL-17 and FAS and FASL Expression

Pearson's correlation analysis showed that the expression of IL-17 mRNA had a significant negative correlation with the expression of FAS and FASL mRNA levels. The higher the IL-17 expression, the lower the FAS and FASL expression and the more severe the LC (*r* = −0.82 for FAS, *r* = −0.847 for FASL, and all *P* < 0.01, Figure 2(b)).

### 3.3. Associations between IL-17 Protein Expression and Clinical Parameters of Patients with LC

The expression of IL-17 in LC tissues was associated with the tumor, node, metastasis (TNM) stage, T stage, lymph node metastasis (LNM), and differentiation (all *P* < 0.05). The results showed that elevated IL-17 expression was observed in the patients with LNM, a low degree of differentiation, late overall TNM stage, and advanced T3/T4 stage, while no significant associations of IL-17 expression were found to be correlated with patient age, gender, and tumor sites (all *P* > 0.05) (Table 2).

### 3.4. Associations between IL-17 Protein Expression and Recurrence in LC Patients

The IL-17 protein expression distributions and associated survival among the patients are shown in Table 3. Among the 60 LC patients, 15 cases were found to have recurrence or metastasis with a median of three years of follow-up. Low IL-17 protein expression was significantly associated with better disease-free survival than the high expression of IL-17 protein (log-rank *P* = 0.022, Figure 2(c)). The ROC curve was constructed for determining the best cut-off value of IL-17 expression, and the area under the ROC curves (AUC) was calculated to evaluate the significant difference of IL-17 expression between LC and adjacent normal tissues. We found that IL-17 expression was significantly associated with the degree of differentiation in the LC patients (AUC = 0.8184, 95% CI: 0.721-0.915, and *P* < 0.0001), and the optimal cut-off value of IL-17 expression was 54.28 (Figure 2(d)). A Cox regression analysis was performed to adjust for other important confounders, including age, sex, tumor size, stage, LNM, and differentiation (Table 3). After adjustment for these confounders, the patients with a high expression of IL-17 had an approximately threefold increased risk of disease recurrence (HR: 3.29, 95% CI: 2.73-8.75) than the patients with a low expression of IL-17, indicating that IL-17 expression was an independent factor for disease influence in patients with LC.

### 3.5. IL-17 Induced FAS and FASL Expression *via* Activation of the PI3K/AKT Pathways

**Figure 3:**
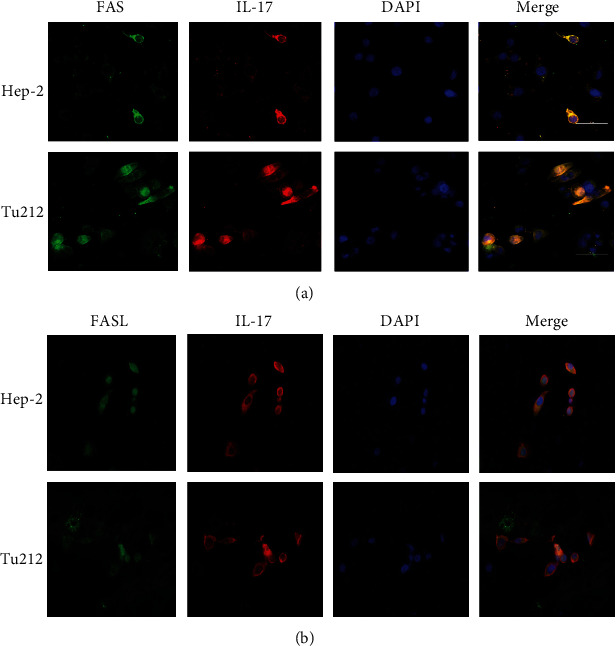
IL-17 colocalized with FAS/FASL in laryngeal carcinoma cells *in vitro*. (a) LC cells were exposed to 50 ng/mL of 200-17 for 6 h and were immunostained with antibodies against FAS (red) and DAPI (blue), followed by confocal microscopy. (b) LC cells were exposed to 50 ng/mL of 200-17 for 6 h, and then they were immunostained with antibodies against FASL (red) and DAPI (blue), followed by confocal microscopy.

**Figure 4:**
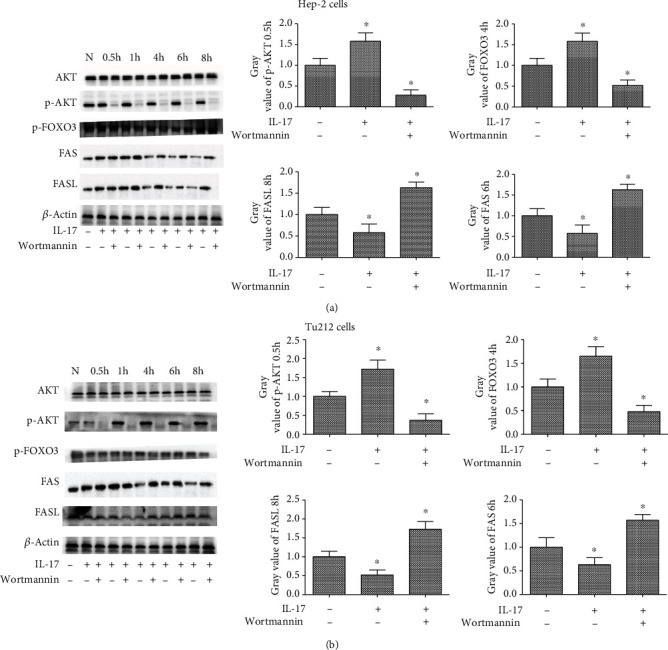
The 200-17-induced FAS and FASL phosphorylation was dependent on PI3K/AKT pathway activation in the LC cell lines. (a) AKT, phosphorylation of AKT, phosphorylated FOXO3, FAS, and FASL, as well as the gray intensity analysis in Hep-2 cells, stimulated with 200-17 for 0.5 h, 1 h, 4 h, 6 h, and 8 h, following pretreatment with wortmannin for 0.5 h. (b) AKT, phosphorylation of AKT, phosphorylated FOXO3, FAS, and FASL, as well as the gray intensity analysis in Tu212 cells, stimulated with 200-17 for 0.5 h, 1 h, 4 h, 6 h, and 8 h, following pretreatment with wortmannin for 0.5 h. *n* = 3 per group. One representative experiment of the three independent experiments is demonstrated. ^∗^*P* < 0.01 versus the control.

To determine whether the IL-17 and FAS/FASL proteins were distributed in the same cellular location, immunofluorescence colocalization experiments were performed using a confocal laser scanning microscope to confirm the localization of IL-17 and FAS/FASL in Hep-2 and Tu212 cells. The confocal microscope analysis revealed that the IL-17 fusion protein colocalized with the FAS/FASL fusion protein in the membrane of Hep-2 and Tu212 cells (Figure 3). *In vitro* Recombinant Human IL-17A (200-17) significantly promoted the expression of IL-17 in LC tissues, which decreased FAS and FASL expression in LC cell lines after a 6 h treatment. However, wortmannin simultaneously abrogated the increase in the phosphorylated PI3K and AKT. The time course for the expression of FAS and FASL in the LC cell lines with stimulation by 200-17 (50 ng/mL) following pretreatment with wortmannin (10 *μ*M/L) was observed. As shown in Figure 4(a), we found that wortmannin simultaneously abrogated the decrease in FAS and FASL induced by 200-17, and the expression of FAS and FASL increased and peaked at 6 h and 8 h after stimulation with 200-17 following pretreatment with wortmannin in Hep-2 cells. Furthermore, wortmannin inhibited the 200-17-elicited upregulation of phosphorylated FOXO3 protein expression. Similar findings were observed in Tu212 cells (Figure 4(b)). Therefore, these results suggested that the activation of the PI3K/AKT pathway may play an important role in the IL-17-mediated reduction of FAS and FASL expressions.

### 3.6. IL-17 Inhibited Apoptosis through the FAS/FASL Pathway in Hep-2 and Tu212 Cells

**Figure 5:**
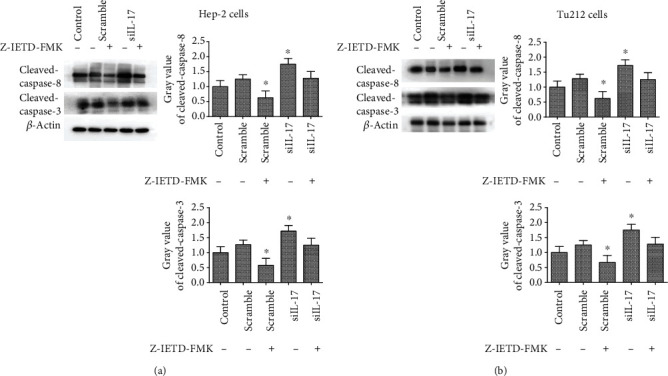
IL-17 inhibited apoptosis through the FAS/FASL pathway in LC cells. (a) The expression levels of cleavage of caspase-8 and cleavage of caspase-3 were examined by Western blotting in Hep-2 cells pretreated with siRNA-IL-17 for 24 h. Following culture for 24 h with or without (40 *μ*M) Z-IETD-FMK for 0.5 h. (b) The expression levels of cleavage of caspase-8 and cleavage of caspase-3 were examined by Western blotting in Tu212 cells pretreated with siRNA-IL-17 for 24 h. Following culture for 24 h with or without (40 *μ*M) Z-IETD-FMK for 0.5 h. All assays were performed in triplicate (^∗^*P* < 0.01 versus the control).

To confirm whether the effects of IL-17 were FAS/FASL-dependent, assays were performed with treatment by Z-IETD-FMK, which is a specific inhibitor of caspase-8. Caspase-8 phosphorylation was inhibited by using Z-IETD-FMK. The protein expression levels of the cleavage of caspase-8 and the cleavage of caspase-3 were evaluated after treatment with 40 *μ*M of Z-IETD-FMK, both with siRNA-IL-17. As shown in Figure 5(a), the Z-IETD-FMK treatment abrogated the siRNA-IL-17-induced promoting cleavage of caspase-8 and cleavage of caspase-3 protein expression in Hep-2 cells. Additionally, the cleavage of caspase-8 and cleavage of caspase-3 protein expression was decreased markedly by the Z-IETD-FMK treatment in the scramble group, while the cleavage of caspase-8/3 did not show lower levels in combination with siRNA-IL-17. Similar findings were observed in Tu212 cells7 (Figure 5(b)). These results indicated that the effect of IL-17 might inhibit apoptosis through the FAS/FASL pathway.

### 3.7. siRNA Silencing IL-17 Gene Expression Promotes Cell Apoptosis

**Figure 6:**
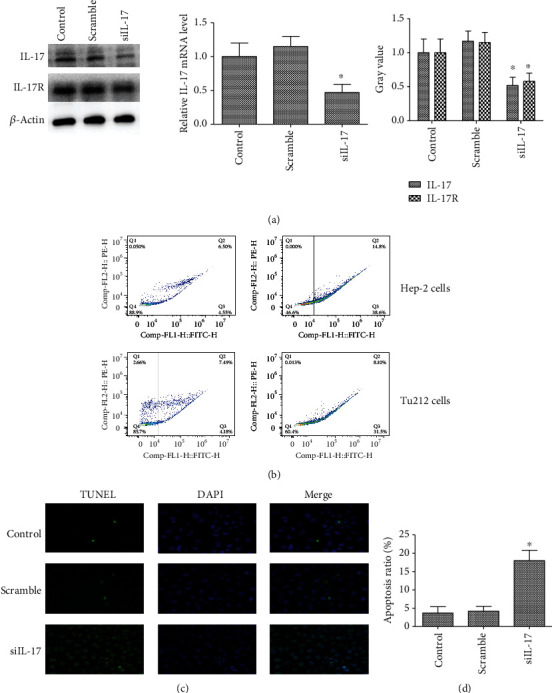
The role of silenced IL-17 in apoptosis of LC cells. (a) Transcriptional expression of IL-17. The real-time PCR revealed that there was a 63% decrease of IL-17 mRNA in LC cells transfected with IL-17 siRNA compared with the control. GAPDH was used as an internal control. One representative experiment of the three independent experiments is demonstrated. ^∗^*P* < 0.01 compared with the control group. The expression of IL-17 in LC cells transfected with siRNA targeting IL-17 was detected with a Western blotting assay. The protein level of IL-17 was reduced to 73% by IL-17 siRNA. Each sample was tested in triplicate. One representative experiment of the three independent experiments is demonstrated. ^∗^*P* < 0.01 compared with the control group. (b) siRNA-IL-17 promoted Hep-2 cell apoptosis (53.4% compared to 11.05% in control cells), while siRNA-IL-17 promoted Tu212 cell apoptosis (39.6% compared to 11.67% in control cells). One representative experiment of the three independent experiments is demonstrated. ^∗^*P* < 0.01 versus the control. (c) siRNA-IL-17 promoted LC cell apoptosis. The TUNEL assay was conducted to detect the apoptotic cells in Hep-2 cells. Representative TUNEL staining results of different groups are shown in a scale bar = 50 *μ*m. (d) The percentages of apoptotic cells in all groups were calculated. Data are presented as mean ± SD. *n* = 7 in each group. Experiments were biologically repeated in triplicate. ^∗^*P* < 0.01 compared to the control group.

To determine the role of IL-17 in LC, the IL-17 gene expression in LC cell lines was silenced using *siRNA* techniques to evaluate the effects of IL-17 on apoptosis in both Hep-2 and Tu212 cells using flow cytometry. The RT-PCR results revealed that there was an approximately 63% decrease in IL-17 mRNA in the stable cell line of the LC cells transfected with the siRNA compared with the controls. In addition, siRNA led to an approximately 73% reduction of IL-17 protein expression according to the Western blotting, as shown in Figure 6(a). Furthermore, it was found that siRNA-IL-17 promoted Hep-2 cell apoptosis (15.0% ± 2.78 compared to 8.20% ± 3.57 in controls). Similarly, siRNA-IL-17 promoted Tu212 cell apoptosis (12.90% ± 4.58 compared to 8.50% ± 2.37 in controls), as shown in Figure 6(b). In addition, apoptotic cells were detected by the TUNEL assay, and siRNA-IL-17 pretreatment dramatically increased the number of apoptotic cells. In addition, staining showed that the number of apoptotic cells in the siRNA-IL-17 group was 3.19 times higher than that of the apoptotic control group (1323.02 ± 278.71 vs. 415.06 ± 68.79, *P* < 0.01), as shown in Figures 6(c) and 6(d). These results proposed that silenced IL-17 expression may be involved in promoting LC cell apoptosis.

### 3.8. siRNA-IL-17 Regulated PI3K/AKT/FAS/FASL Pathways in Hep-2 Cells and Tu212 Cells

**Figure 7:**
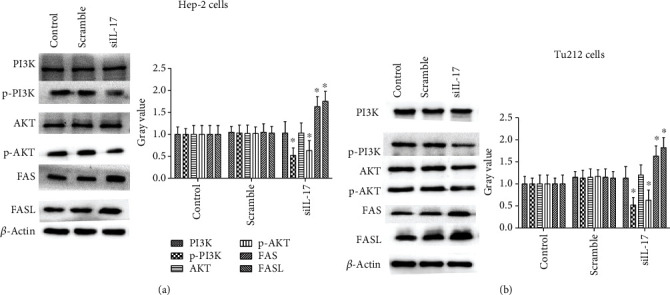
Effects of silencing LC cell IL-17 gene expression on the activation of PI3K/AKT/FAS/FASL pathways in the LC cell lines. (a) Downregulation of the IL-17 gene expression by siRNA causes expression of FAS and FASL to increase activation in Hep-2 cells. (b) Downregulation of the IL-17 gene expression by siRNA causes expression of FAS and FASL to increase in Tu212 cells. One representative experiment of the three independent experiments is demonstrated. ^∗^*P* < 0.01 compared with the control group.

To further examine the mechanism by which siRNA-IL-17 induced apoptosis, the protein levels of PI3K, P-PI3K, AKT, P-AKT, FAS, and FASL were detected using Western blotting in the siRNA-IL-17-transfected LC cell line and the controls. P-PI3K and P-AKT activities were significantly inhibited by the IL-17 knockdown in the Hep-2 cells and Tu212 cells, while FAS and FASL were markedly elevated in the IL-17 siRNA group compared with the controls, as shown in Figure 7. These results indicated that the silencing of IL-17 expression mediated apoptosis *via* the PI3K/AKT/FAS/FASL pathways in LC cells.

## 4. Discussion

LC is one of the most common malignant head and neck tumors, accounting for 1–2.5% of all malignancies throughout the body [28, 29]. With advancements in the treatment and management of LC, the prognosis has significantly improved. However, the survival of this tumor still remains poor, with a five-year overall survival of less than 60%. In particular, the prognosis for advanced stage and recurrent tumors of this disease are still poor after treatment with surgery and chemoradiotherapy. Additionally, the efficacy of chemoradiotherapy still remains limited [30, 31]. The clinical data of patients in this study showed that cytokines are dysregulated in LC tissues as compared with normal tissues. Our previous study also found that well-differentiated tumor tissue had high mRNA expression levels of IL-12R*β*2 and INF-*γ*, whereas moderately and poorly differentiated tumor tissues had low levels of these markers [32]. COL7A1-UCN2 had the highest frequency in LC tissues (13/23; 56.5%). Furthermore, COL7A1-UCN2 positivity was significantly associated with the overall survival of LC patients [33]. Previous studies have reported that patients with early-stage laryngeal carcinoma exhibited a lower level of IL-17 mRNA expression than those with advanced stages, and cancer tissues exhibited a significantly higher level of IL-17 mRNA expression than pericarcinoma tissues [34, 35]. The results from this current study may support the theory that targeting IL-17 may be a therapeutically useful treatment avenue for LC.

In this study, the effects of IL-17 on apoptosis of LC cells and the role of IL-17 acting as a biomarker in the recurrence of this disease, metastasis, and prognosis of LC patients were explored. The tumor microenvironment is infiltrated by a wide array of cells from both the adaptive and innate immune systems, and the hematoxylin and eosin assays have demonstrated increased lymphocytic infiltration, with a decrease in the degree of differentiation in laryngeal carcinoma. We found that IL-17 expression increased in different pathological grades of LC tissues compared with paracancer tissues of the normal controls in both levels of mRNAs and proteins. We found that the expression of IL-17 and IL-17R mRNA in the tumor group was significantly higher than that in the control group. These findings were consistent with those levels of protein expression. The levels of expressions of the IL-17, IL-17R, P-AKT, and P-PI3K proteins significantly increased with increasing severity of LC, while the expression of FAS and FASL significantly decreased with increasing severity of LC. Previous studies have reported that the patients with early-stage laryngeal carcinoma exhibited a lower level of IL-17 mRNA expression than those with advanced stages, and cancer tissues exhibited a significantly higher level of IL-17 mRNA expression than pericarcinoma tissues [34, 35]. Overexpression of both IL-17 and IL-17R was observed in tumors compared with adjacent tissues [36, 37]. Furthermore, our results showed that IL-17 and IL-17R expression in LC tissue was correlated with tumor size, TNM stage, degree of differentiation in LC, and LNM. These results demonstrated that the expression of IL-17 was a significantly independent factor that may contribute to the recurrence of LC patients after adjustment with other significant confounders, including tumor size and TNM stage [38–40]. A previous study also demonstrated that increased IL-17 expression had a correlation with poor overall survival in other types of cancers [41].

FAS and FASL are members of the tumor necrosis factor (TNF) receptors and TNF family, respectively. The ligation of FAS with FASL results in the activation of a caspase cascade that initiates apoptosis [42–44]. With the aggravation of LC, the expression of FAS and FASL in LC tissues markedly decreases, suggesting that suppression of the FAS/FASL pathway could be caused by the inhibition of apoptosis in LC cells. We found in this study that mRNA levels of FAS and FASL increased in cancer tissues compared to the control with the increasing severity of LC, which was not consistent with the protein expression. Several reports have shown that the relationship between mRNA and protein is not always strictly linear but has a more intrinsic and complex dependence. Different regulatory mechanisms (such as synthesis and degradation rates) may play roles in both the synthesized mRNA and the synthesized protein [45–47]. In this study, the data showed that IL-17 expression had a negative correlation with the expression of FAS and FASL. A previous study highlighted IL-17 to be a potential prognostic marker for patients with respiratory, digestive, and other system cancers [13]. In addition, IL-17 may be significantly correlated with the differentiation and angiogenesis in the development of LC as the expression of CXCL9, CXCL10, and IL-17 mRNAs in the skin of FAS- and FASL-deficient mice was decreased [27, 36, 37]. In this study, the IL-17 fusion protein colocalized with the FAS/FASL fusion protein in the membrane of Hep-2 and Tu212 cells. IL-17 activates the PI3K/AKT signaling pathway, which may block the FAS-associated death domain protein, and this inhibits the cellular apoptosis pathway by improving the phosphorylation of FOXO3a [48, 49]. Recombinant Human IL-17A (200-17) had a growth-stimulating effect on IL-17 [50, 51]. Wortmannin covalently modifies PI3K and is a potent and specific PI3K inhibitor [52–54]. The results of this study showed that the expression of FAS and FASL increased after stimulation with the 200-17 following pretreatment with wortmannin in Hep-2 cells and Tu212 cells. Furthermore, wortmannin inhibited 200-17-elicited upregulation of phosphorylated FOXO3 protein expression. Among cell death receptors, the FAS/FASL system provides an important apoptotic mechanism [52, 55]. FAS-mediated apoptosis following death receptor stimulation leads to cleavage of procaspase-8 and subsequent activation of a downstream caspase, such as caspase-3, which induces apoptosis [56, 57]. Cells treated with siRNA-IL-17 and Z-IETD-FMK showed a greater decrease in the cleavage of caspase-8 and the cleavage of caspase-3 protein expression compared with treatment with siRNA-IL-17 alone. These results suggested that IL-17 stimulation might restrain apoptosis *in vitro* by activating the PI3K/AKT pathway, subsequently leading to inhibition of the FAS/FASL pathway.

Many studies have demonstrated that IL-17 acted as a tumor promoter and enhanced cell proliferation, migration, and invasion [58, 59]. However, little has been reported regarding the mechanism by which IL-17 is involved in the progression of LC. To further investigate the role of IL-17 in the mitogenic effects on LC cells, in the present study, endogenous IL-17 was silenced by siRNA to determine the effect of endogenous IL-17 in LC cells. We found that the downregulation of IL-17 expression inhibited the PI3K/AKT pathway, which in turn significantly evaluated the activation of the FAS/FASL pathway, leading to enhanced apoptosis of LC cells. These findings might indicate that silenced IL-17 expression might significantly promote LC cell apoptosis *via* the PI3K/AKT/FAS/FASL pathways.

## 5. Conclusions

In summary, the present investigation provides further evidence for the involvement of IL-17 in the progression of LC. The aberrant expression of IL-17 contributes to the pathogenesis of LC. Interference of the expression of IL-17 at the posttranscriptional level could enhance the apoptosis of LC cell lines *via* activation of the FAS/FASL pathway, suggesting that IL-17 can be targeted for therapeutic intervention against LC. Further studies are required to elucidate the mechanisms and diverse signaling networks of IL-17 related to LC.

## Figures and Tables

**Table 1:** Primer sequences of IL-17, IL-17R, FAS, FASL, and GAPDH.

Primers used	Forward	Reverse
IL-17	5′-GACCCTTCACCCCTCACC-3′	5′-TTATGGATCATGCCCACAAG-3′
IL-17R	5′-GGGATTACAGGCGTGAGCCA-3′	5′-GCGGTCTGGTTATCGTCTAT-3′
FAS	5′-GGTGCTGTCTCTCTATGCCTCTGGA-3′	5′-GGTGCTGTCTCTCTATGCCTCTGGA-3′
FASL	5′-AAGGACCTCCAGCATCACTGTGTCA-3′	5′-CCTTCAGAGCCCGCAGCTTCCACGT-3′
GAPDH	5′-AAAGTCCGCCATTTTGCCACT-3′	5′-CCAAATCGTTAGCGCTCCTT-3′

**Table 2:** Correlations of IL-17 expression with clinical characteristics in patients with LC.

Clinical characteristics		*n*	IL-17 expression	*P*
Gender				0.280
	Male	35	3.89 ± 3.28	
	Female	25	4.28 ± 3.42	

Age				0.321
	<50	22	5.48 ± 2.74	
	≥50	38	4.29 ± 3.37	

TNM stage	I/II	27	4.82 ± 0.53	0.007
	III/IV	33	5.36 ± 3.24	

Degree of differentiation				0.009
	Middle/high	42	4.58 ± 3.27	
	Low	18	6.17 ± 3.37	

LNM				<0.0001
	Yes	38	5.63 ± 3.16	
	No	22	3.15 ± 2.37	

Tumor site				0.285
	Glottis	26	3.47 ± 2.31	
	Supraglottis	20	2.25 ± 3.43	
	Subglottis	4	3.54 ± 3.22	

T classification	Tis	8	2.04 ± 4.13	0.013
	T1/T2	23	3.61 ± 4.13	
	T3/T4	29	4.75 ± 2.36	

LC: laryngeal cancer; TNM: tumor node metastasis; LNM: lymph node metastasis.

**Table 3:** Association of IL-17 expression with disease recurrence among the patients with LC (*n* = 60).

IL-17 expression	Recurrence number/all patients' number	aHR^1^ (95% CI)	*P*
Low^2^	8/38	1.00	
High	7/22	3.29 (2.73-8.75)	0.028

^1^Hazard ratio adjusted for age, sex, tumor size, TNM stage, LNM, and differentiation. ^2^Reference group (the median of expression as a cut-off point in patients without recurrence). LC: laryngeal cancer; TNM: tumor node metastasis; LNM: lymph node metastasis.

## Data Availability

All data generated or analyzed during this study are included in this published article. The datasets used and/or analyzed during the current study are available from the corresponding authors upon reasonable request.

## Abbreviations

IL-17: Interleukin-17
siRNA: Small interfering RNA
RT-qPCR: Reverse transcription-quantitative polymerase chain reaction
LNM: Lymph node metastasis.
